# Case report: restored vision after ocular Closantel intoxication and blindness

**DOI:** 10.1186/s12886-021-01916-4

**Published:** 2021-03-31

**Authors:** Mohammad Reza Khalili, Athar Zareei

**Affiliations:** Poostchi Ophthalmology Research Center, Shiraz University of Medical Sciences, Zand Street, Shiraz, Iran

**Keywords:** Closantel, Toxicity, Electroretinography, Case report, Vision, Ocular

## Abstract

**Background:**

Closantel is the best-known anti-parasitic medicine for veterinarians, which is contraindicated in humans. After reviewing the literature on ocular toxicity following mistaken usage of Closantel in humans, this report was found as the first complete restoration of visual function after Closantel intoxication. This report could be useful in anticipating the possibility of a further improvement based on a dose-response relationship. An important point of this report is the apparent reversibility of the vision and Electrophysiological parameters after Closantel intoxication and blindness. To conclude, the present case report demonstrates the importance of immediate referral and management in Closantel intoxication to avoid the long-term adverse effects of drug on visual function.

**Case presentation:**

A 47-year-old man mistakenly took about 20 cc of Closantel 5% (15.87 mg/kg). Four hours after mistaken usage of Closantel, he was transferred to the district hospital due to dizziness and nausea. His stomach was washed out immediately after hospital arrival. He was being hospitalized in that hospital for 3 days. Then, he was referred to our clinic due to progressive vision loss. Methylprednisolone acetate 250 mg was injected once on 5th day after taking Closantel. His vision was reducing gradually so low that he could only detect hand motion (HM) on the 14th day after taking Closantel. ERG test was requested. It showed an exclusive reduction in b-wave amplitude under photopic and scotopic conditions. Later, his vision surprisingly improved gradually and his visual acuity was fully restored on the 28th day after the incident. After 3 years, we checked him again. His visual acuity was 20/20 in both eyes and the patient did not have any problem and his ERG report was completely normal.

**Conclusions:**

In low dose of Closantel and immediate referral, ocular toxicity could be resolved.

## Background

Closantel is the best-known Halogenated Salicylates, which is mainly used to treat parasitic infections in animals and has been contraindicated in humans. Accidental usage of Closantel in humans causes ocular toxicity. We report the first complete restoration of visual function after Closantel intoxication. This report could be useful in anticipating the possibility of a further improvement based on a dose-response relationship. Herein, we present a patient who unintentionally consumed this drug and had transient blindness for a month. An important point of this report is the apparent reversibility of the vision and Electrophysiological parameters after Closantel intoxication and blindness. Full restoration of visual function appears due to immediate gastric lavage at 4 h after taking Closantel.

## Case presentation

A 47-year-old man had referred to our center complaining of gradual vision loss. During history taking, it became evident that the patient had mistakenly took Closantel syrup instead of stomach medicine Alum-Mag. The volume of consumed syrup was measured about 20 cc. Each milliliter of the syrup contains 50 mg of Closantel 5% (1000 mg closantel/15.87 mg/kg). Patient’s weight was 53-Kg. The patient remarked that 4 hours after taking Closantel, he was transferred to the district hospital due to dizziness and nausea. His stomach had been flushed out immediately after hospital arrival. He had been hospitalized for 3 days. He was then referred to our clinic due to the progressive vision loss.

**Fig. 1:**
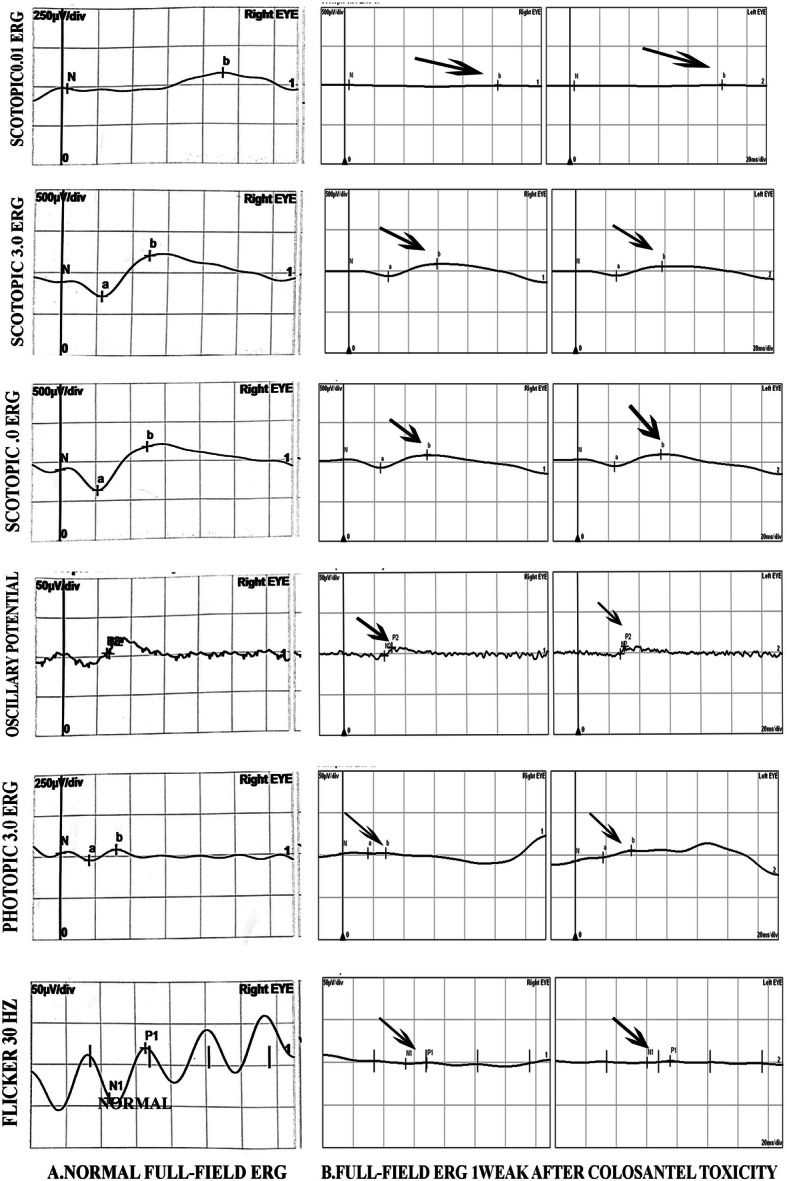
a: Normal ERG responses and patient’s ERG responses 1 week after Closantel ocular toxicity. All six standard in the normal person of the same age which obtained with the same instrument(**a**) and all six standard ERG respons of the patient showed remarkable declines in both eyes(**b**)

**Fig. 2:**
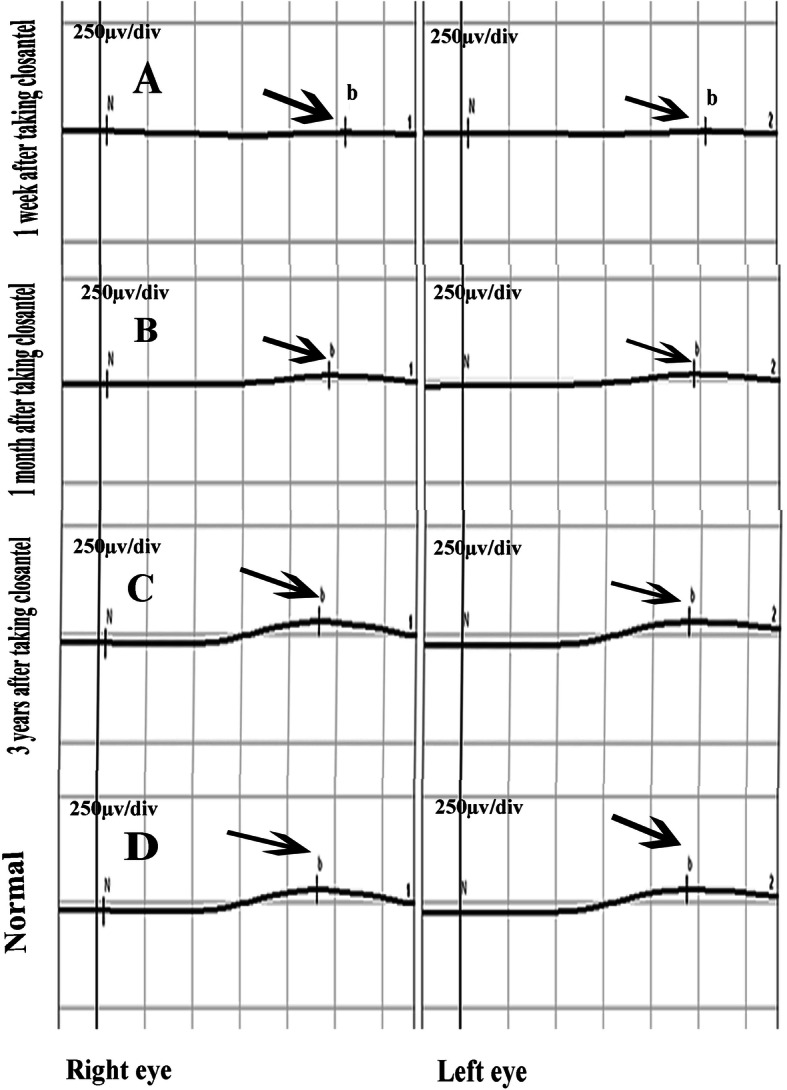
The apparent improvement in Scotopic ERG responses in three different times after taking closantel and a normal scotopic ERG response in both eyes. Scotopic recorded 1 week after Closantel toxicity(**a**),Scotopic ERG recorded 1 month after Closantel toxicity(**b**), Scotopic ERG response 3 years after Closantel toxicity(**c**), normal Scotopic ERG in the normal individual of the same age with the same instrument(**d**)

**Fig. 3:**
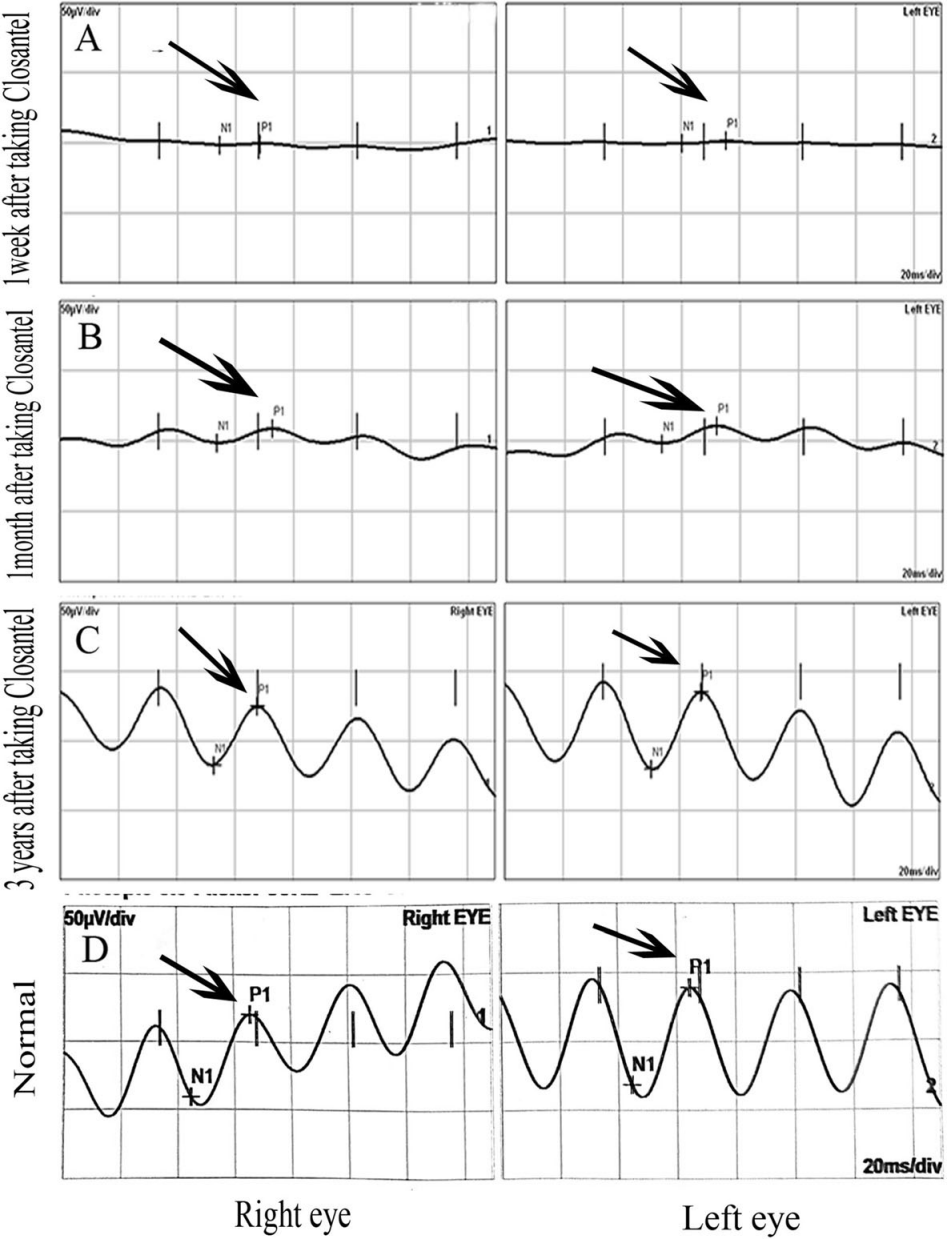
The apparent improvement in Photopic ERG responses (3.0 Flicker 30 Hz) ERG in three different times after taking closantel and a normal Photopic ERG response in both eyes. Photopic ERG responses 1 week after Closantel toxicity(**a**), Photopic ERG responses recorded 1 month after Closantel toxicity(**b**), Photopic ERG responses recorded 3 years after Closantel toxicity(**c**) and normal Photopic responses in the normal individual of the same age with the same instrument(**d**)

**Fig. 4:**
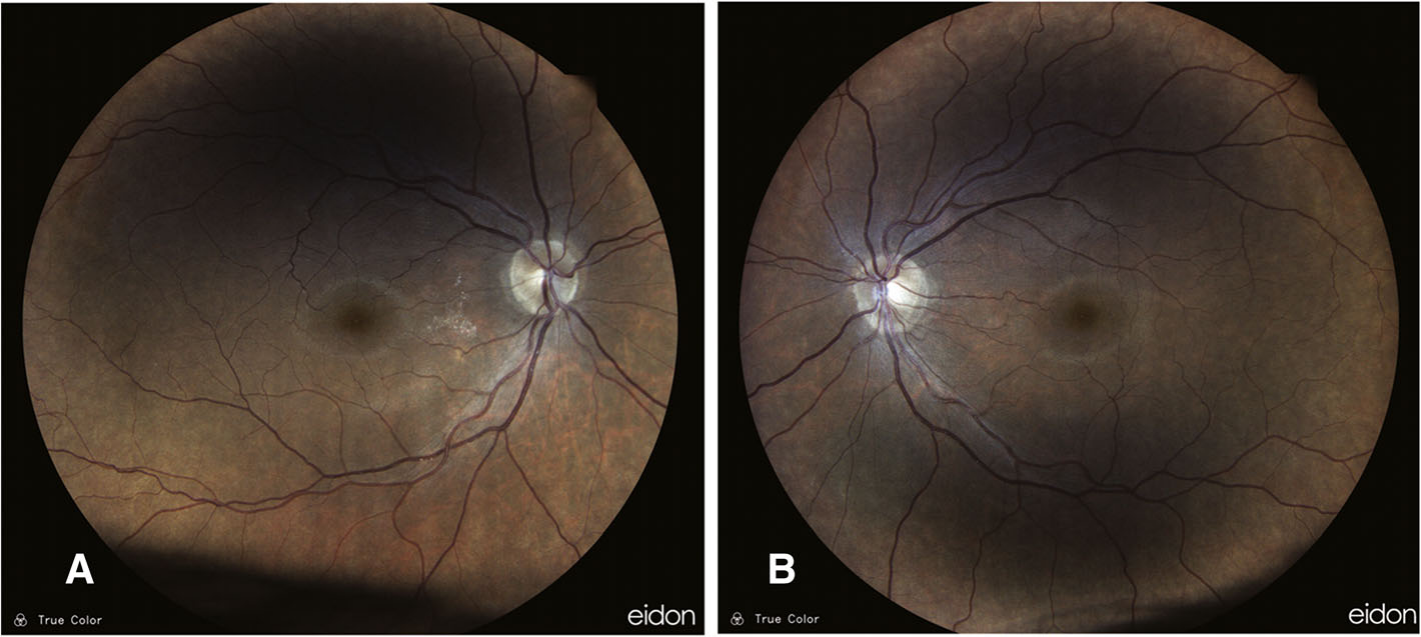
Fundus photography after restored vision during the follow-up examination. Right eye (**a**), Left eye (**b**)

**Fig. 5:**
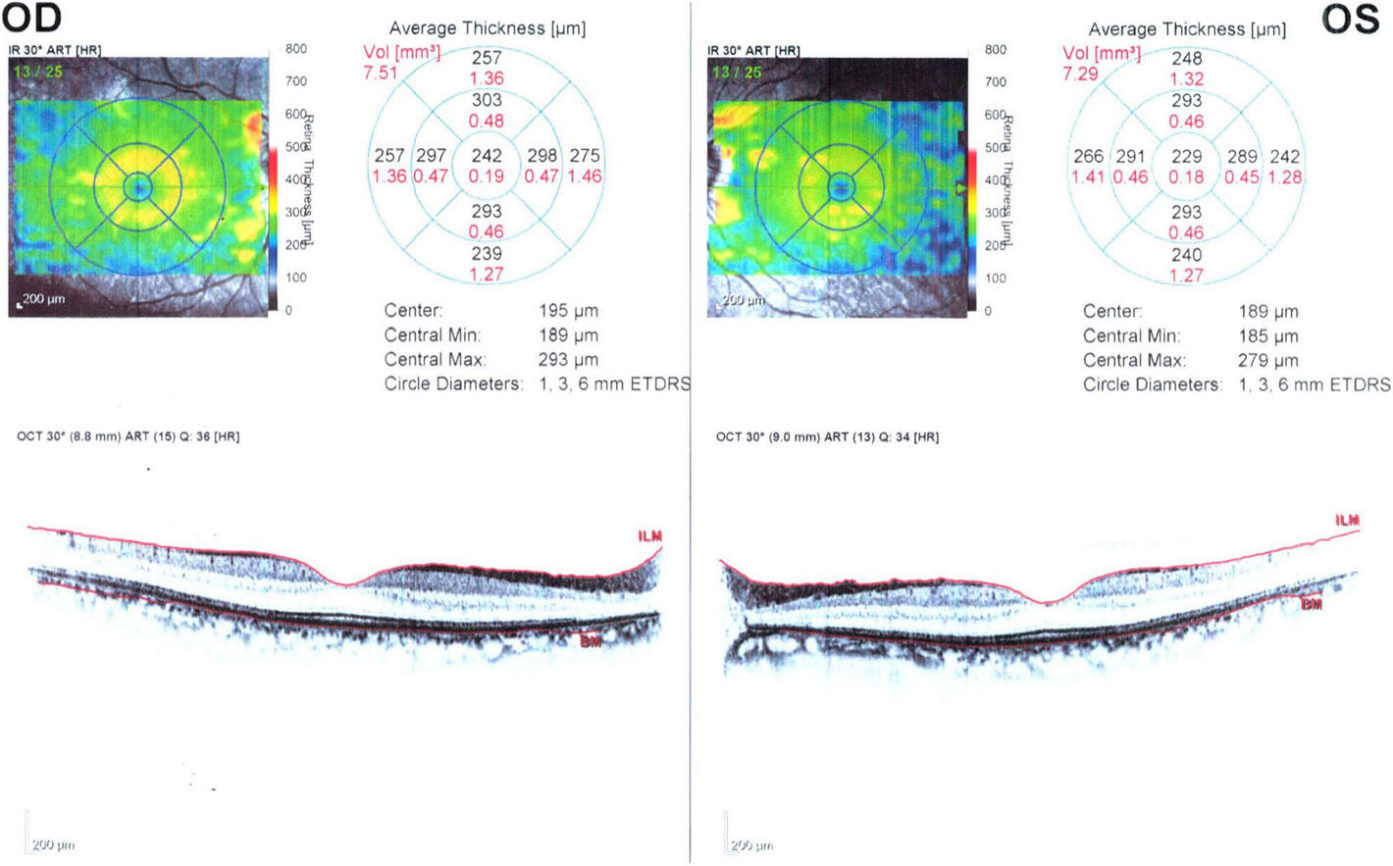
Macular OCT after restored vision during the follow-up examination. Right eye (OD), Left eye (OS)

His visual acuity was 20/200 for both eyes at the time of arrival to our clinic and intraocular pressure was 18 mmHg and 16 mmHg in the right and left eye respectively (measured using Goldman applantation tonometer). There was no pathologic sign during slit lamp examination and refractive error of the patient was not significant. In fundus examination, mild optic disc swelling was observed in both eyes 250 mg. Methylprednisolone acetate was injected intravenously once on the 5th day after taking Closantel. His Vision was reducing gradually so low that he could hardly detect hand motion (HM) at 0.5 m on the 14th day after taking Closantel. ERG test was requested. ERG test performed according to electrophysiological standards. The six basic ERGs defined by the ISCEV Standard. The full-field electroretinogram (ERG) is an electrophysiologic test that shows retinal function in the light and dark phases. The full-field ERG results of this patient showed remarkable decline of all ERG steps compared to normal (Fig. 1). ERG records compared with those of a normal person of the same age which obtained with the same instrument. A significant decline in b-wave amplitude and a significant increase in implicit time were seen under scotopic condition (Scotopic 0.01 ERG GF). Remarkable decline in the amplitude of the flicker ERG under photopic condition was also found (Photopic 3.0 Flicker 30 Hz ERG GF). Systemic examination and brain imaging did not show any specific results. Patient’s medical history was negative for other ocular and systemic diseases. Later, his vision surprisingly improved gradually and his visual acuity was 20/20 and fully restored on the 28th day after the incident. Again, an ERG test was requested. Scotopic and photopic ERG test were significantly improved, but not within the normal range. As shown in Figs. 2 and 3, b-wave amplitude and latency under scotopic condition and oscillatory potential under photopic condition were getting better but they were still different from normal ERG recording. Afterwards the patient was asymptomatic. After 3 years, we examined him again. His visual acuity was 20/20 in both eyes and his Full-field ERG showed remarkable improvement under both scotopic and photopic conditions and was as the same normal individual. ERG recordings of the patient and ERG recordings of the same age normal individual under scotopic and photopic conditions are shown in Figs. 2 and 3 in the order of recording time. We requested visual field test. Since the patient was illiterate, his cooperation was so weak, and his Humphrey Visual Field was unreliable. However, his confrontation visual field was normal. There were no suspicious signs in the retina during indirect ophthalmoscopy and his fundus was normal. Fundus examination was normal (Fig. 4). During the follow-up, the macular OCT images were taken that was normal (Fig. 5).

## Discussion and conclusions

Closantel has a broad-spectrum anti-parasitic effect for veterinarian usage. Closantel’s pharmacokinetics are unclear in humans. Proposed oral doses in sheep and cattle were 7.50-10 mg/kg and 10-15 mg/kg respectively [1]. Three times this amount is considered Closantel intoxication in animals. Our patient took 15.87 mg/kg. There have been several reports of ocular Closantel toxicity in humans (Table 1). The first report was 11 Lithuanian women who lost their eyesight after consuming Closantel in 1993. The authors assessed some of these reported patients 17 years after poisoning for the assessment of late ocular changes after Closantel poisoning and indicated lasting damage of the optic nerve and retina [2]. These patients were reexamined and reported 22 years after poisoning and demonstrated long lasting negative effects of the medication on the retina, with no significant recovery [3]. Other reports have demonstrated that Closantel consumption often leads to blindness [4–7] except for one case that was treated with a proposed therapeutic approach (Plasma Exchange in 5 sessions); however, in that case the final visual acuity was reported 20/25 and there was residual central amplitude impairment in multifocal ERG [8]. Among previous reports, the least time between the first use of Closantel and the clinic referral was 1 day, but it was 4 h in our patient.

**Table 1:** Previous case reports and case series of closantel toxicity (1993–2020)

Patients Gender (Age)	Author (Year of reporting)	Cumulative dose	time	treatment	Final VA	Clinical assessment and Paraclinical tests
F(3)	Badrane et al. (2013)	N/A	24 h	Oral vitamins b1, b6,b12	blindness	bilateral mydriasis no pupillary reflex severe papilloedema radiological investigations:normal biological exams:normal
M (40) M (44)	Badrane et al. (2013)	N/A	One week One week	Unknown	blindness	Fundos photograph: normal visual feld: scotoma Laboratory tests: normal
F(5)	Essbar et al. (2014)	500 mg/day for 8 day	8 days	Glucocorticoids, vitamin B12,vitamin K correction of blood, coagulation parameters,normalization of liver enzymes	blindness	bilateral mydriasis,no pupillary reflex,papilledema ERG: decreased retinal activity VEP: decreased amplitude and delayed peak latency hepatic enzymes: acute increase leukocytosis anemia coagulopathy
M(50)	Koziolek et al. (2015)	2700 mg of mebendazol,1800 mg of closantel	7 days	Plasma Exchange in 5 sessions	OU: Hand movements (before treatment) OU: 20/25	VF: markedly constricted at outer margins VEP: decreased amplitude and delayed peak latency ERG:decreased photoreceptor signal amplitude and normal latency After treatment: mfERG: showed residual central amplitude impairment.
M(34)	Tabatabaei et al. (2016)	three 500 mg tablets	10 days	injections 20,000 units daily for 3 days 1 mg/kg oral prednisolone 1 g intravenous methylprednisolone acetate for 3 days	no light perception (NLP)	macular OCT: disruption in ORL outer retina ERG: severe decreased photopic and scotopic respons VEP: decreased amplitude and delayed peak latency serum hemoglobin, 11 mg/dl ALT: increase more than two times AST:increase more than two times
F(28) F(22) F(25) F(38) F(24)	Hoen et al. (1993) Asoklis et al. (2015) Asoklis et al. (2018)	3 tablets 3 tablets 3 tablets 3 tablets 3.5 tablets	N/A	N/A	OD:20/63 OS: 20/200 OD:20/25 OS: 20/40 OD: 20/20 OS: 20/20 OD: 20/40 OS: 20/40 OD: 20/25 OS: 20/25	The last examination reported: All patients reported Limited visual function VF: markedly constricte fundos photography: changes in the fundus VEP: decreased amplitude and delayed peak latency SD-OCT: thinner retinal pigment epithelium, photoreceptor وouter nuclear, outer plexiform, and inner nuclear retinal layers

*F* Famale, *M* Male, *Time* The time between taking Closantel and starting treatment, *OCT* optical coherence tomography, *ERG* Electroretinogram, *mfERG* multifocal Electroretinogram, *VEP* visual evoked potential, *ALT* liver aminotransferases (alanine), *AST* aspartate aminotransferase, *SD-OCT* spectral domain optical coherence tomo-graph

The Closantel pharmacokinetics in humans are unknown [8]. Closantel toxicity in animals show that myelinic oedema leads to acute swelling of the optic nerves. It results in compression within the bony optic canals and leads to fibrosis of the nerve segments and consequent necrosis [9]. An acute degenerative change was seen in the outer retinal layers, that could not be secondary to the optic neuropathy, because retrograde transsynaptic degeneration of the photoreceptor neurons is not found, even when the optic nerve is completely transected, so the optic neuropathy and retinopathy are separate toxic effects [4, 9].

An important point of the present case is the apparent reversibility of the vision and ERG parameters that could be due to a single low-dose of Closantel and rapid gastric emptying. The specific cause of visual restoration may not be determined by certain, even though these can be acceptable. This is the first report that signifies restored vision after ocular Closantel intoxication and blindness. To conclude, the present case report demonstrates the importance of immediate referral and management in Closantel intoxication to avoid the long-term adverse effects of drug on visual function.

## Data Availability

The datasets used during the current study are available from the corresponding author on reasonable request.

## Abbreviations

ERG: Electroretinogram
HM: Hand motion
ERG GF: Electroretinogram ganzfeld

## References

[1] Joint F, Additives WECoF, Organization WH (2004). Evaluation of certain veterinary drug residues in food: sixty-second report of the Joint FAO/WHO expert committee on food additives: world health organization.

[2] Asoklis R, Augyte A, Cimbalas A. Late ocular changes after closantel (Flukiver) poisoning. Acta Ophthalmol. 2015;93. 10.1111/j.1755-3768.2015.0436.

[3] Asoklis R, Cimbalas A, Augyte A, Jasinskiene E, Strupaite R. Late ocular changes after closantel poisoning in five women. Eye. 2018;32(12):1800–2. 10.1038/s41433-018-0180-6 PMC6292853 30076367

[4] Huxtable C, Dorling P, Slatter D (1980). Myelin oedema, optic neuropathy and retinopathy in experimentalstypandra imbricata toxicosis. Neuropathol Appl Neurobiol.

[5] Badrane N, Abbada A, Chaoui H, Aoued L, Rhalem N, Benjelloune BS (2013). Blindness following closantel poisoning: Report of three cases. Clinical toxicology.

[6] Essabar L, Meskini T, Ettair S, Erreimi N, Mouane N (2014). Harmful use of veterinary drugs: blindness following Closantel poisoning in a 5-year-old girl. Asia Pac J Med Toxicol.

[7] Tabatabaei SA, Soleimani M, Mansouri MR, Mirshahi A, Inanlou B, Abrishami M, Pakrah AR, Masarat H (2016). Closantel; a veterinary drug with potential severe morbidity in humans. BMC Ophthalmol.

[8] Koziolek MJ, Patschan D, Desel H, Wallbach M, Callizo J (2015). Closantel poisoning treated with plasma exchange. JAMA Ophthalmol.

[9] Gill P, Cook R, Boulton J, Kelly W, Vanselow B, Reddacliff L (1999). Optic neuropathy and retinopathy in closantel toxicosis of sheep and goats. Aust Vet J.

